# Genetic Evaluation of Hip Score in UK Labrador Retrievers

**DOI:** 10.1371/journal.pone.0012797

**Published:** 2010-10-22

**Authors:** Thomas W. Lewis, Sarah C. Blott, John A. Woolliams

**Affiliations:** 1 Kennel Club Genetics Centre at the Animal Health Trust, Animal Health Trust, Newmarket, United Kingdom; 2 The Roslin Institute and Royal (Dick) School of Veterinary Studies, University of Edinburgh, Roslin, United Kingdom; Ohio State University Medical Center, United States of America

## Abstract

Hip dysplasia is an important and complex genetic disease in dogs with both genetic and environmental influences. Since the osteoarthritis that develops is irreversible the only way to improve welfare, through reducing the prevalence, is through genetic selection. This study aimed to evaluate the progress of selection against hip dysplasia, to quantify potential improvements in the response to selection via use of genetic information and increases in selection intensity, and to prepare for public provision of estimated breeding values (EBV) for hip dysplasia in the UK. Data consisted of 25,243 single records of hip scores of Labrador Retrievers between one and four years old, from radiographs evaluated between 2000 and 2007 as part of the British Veterinary Association (BVA) hip score scheme. A natural logarithm transformation was applied to improve normality and linear mixed models were evaluated using ASREML. Genetic correlations between left and right scores, and total hip scores at one, two and three years of age were found to be close to one, endorsing analysis of total hip score in dogs aged one to three as an appropriate approach. A heritability of 0.35±0.016 and small but significant litter effect (0.07±0.009) were estimated. The observed trends in both mean hip score and mean EBV over year of birth indicate that a small genetic improvement has been taking place, approximately equivalent to avoiding those dogs with the worst 15% of scores. Deterministic analysis supported by simulations showed that a 19% greater response could be achieved using EBV compared to phenotype through increases in accuracy alone. This study establishes that consistent but slow genetic improvement in the hip score of UK Labrador Retrievers has been achieved over the previous decade, and demonstrates that progress may be easily enhanced through the use of EBVs and more intense selection.

## Introduction

It has been argued that tackling the spread of heritable diseases in managed populations of animals is an obligation for animal breeding, both for farm and for companion animals [1]. Among companion animals the extent of inherited disease in pedigree dog breeds has recently come under scrutiny [2], [3] and it has been reiterated that the appropriate use of genetic selection schemes could be used to tackle such diseases [4]. Thus, researchers have a responsibility to make the best use of the data available to provide the tools that will enable breeders to reduce the disease burden across pedigree dog breeds through effective and efficient genetic selection.

Hip dysplasia is an important and complex genetic disease in dogs, and is one of the most prevalent diseases among larger breeds [5]. It has both genetic and environmental influences [6], [7], [8] with evidence of gene effects at multiple loci [9] confirming complex underlying genetics. It is a developmental orthopaedic disorder characterised by the formation of a loose, ill-fitting coxofemoral (hip) joint [10], and over time, the malformation leads to abnormal wearing of bone surfaces and the appearance of the osteoarthiritic signs of degenerative joint disease (DJD) [11]. Since the osteoarthritis that develops is irreversible, hip dysplasia is often impossible to treat and so the only way to improve dog welfare, through reducing the prevalence, is through genetic selection.

Various international recording schemes for hip dysplasia have been in existence for several years with a view to providing information to breeders. One of the larger and longer running schemes is the British Veterinary Association (BVA) / Kennel Club (KC) hip scoring scheme, which was established in its current form in 1984 [12] and is used in the UK, Ireland, Australia and New Zealand. Under this scheme, a radiograph of the pelvic area is taken by a general practice vet according to standardised protocols, submitted to the BVA and evaluated by three members of a panel of certified radiologists or small animal surgeons [13]. Nine features of each hip are scored according to the degree of dysplasia observed (0 = no signs) and scores from each feature are summed to give a total from 0–53 for each hip, and 0–106 in total. Although voluntary, the rate of participation in the UK is good; for example, 8–10% of all annually registered Labrador Retrievers (the most popular breed in the UK, with between 33, 398 registrations in 1999 and 45,779 registrations in 2005) are scored per year, equating to 50–60% of all dogs used for breeding. There are other international evaluation schemes of note, operated by the Orthopedic Foundation for Animals (OFA) in the USA and Canada; the Fédération Cynologique Internationale (FCI), an umbrella organisation of more than 80 national kennel authorities in most of Europe, Russia, South America and parts of Asia, [13]; and the PennHIP scheme based at the University of Pennsylvania, USA. There are clear philosophical differences between these global recording schemes in the best way to assess hip dysplasia, particularly over categorical classification of the disease and whether an assessment is made on the condition of both hips or on the worst hip alone.

Reports on the response to selection against the disease are mixed. In the UK, it was reported that there were no discernable improvements in 5 breeds between 1987 and 1995 [14], despite the availability of hip score phenotypes to breeders. There have been reported reductions in the prevalence of disease in some Finnish registered breeds but an increase in others, even when an enforced threshold of hip score was applied to registrations [15]. An improving genetic trend with respect to disease was detected in two Swedish registered breeds [16] and in a retrospective study of data on American Labradors over almost 40 years [17], via calculation of estimated breeding values (EBV). These inconsistencies lead to the impression that progress in breeding away from the disease is currently sub-optimal given the amount of information being generated.

The complex nature of hip dysplasia, the abundance of phenotypic data and the availability of pedigree records are favourable for the calculation of EBV for the scored features indicative of this disease. EBV are the estimates of genetic liabilities obtained after removing as far as possible the environmental influences. The calculation of an EBV combines an individual's phenotypic data with that of its relatives using weights derived from the pedigree, resulting in a more accurate estimate than is possible from using only the individual's own phenotype. EBV have been widely used in the improvement of production traits and traits indicative of health in livestock [18], [19], and have more recently been calculated for complex inherited diseases in dogs [16], [20], [21].

Given the mixed reports of the response to selection against hip dysplasia and the suitability for selection using EBV, this study aimed to assess one of the largest cohorts of data on a single breed (Labrador Retrievers) within the BVA/KC scheme. The objectives were to: 1) evaluate the current effectiveness of the scheme in enabling demonstrable and genetic improvement towards reducing prevalence; 2) quantify the potential benefits from providing better genetic information such as EBV to breeders; 3) prepare the path for routine provision of EBV with the BVA/KC data. These objectives were carried out using both mixed model analysis of the accumulated data of BVA/KC and simulation.

## Results

### Data distribution

**Figure 1:**
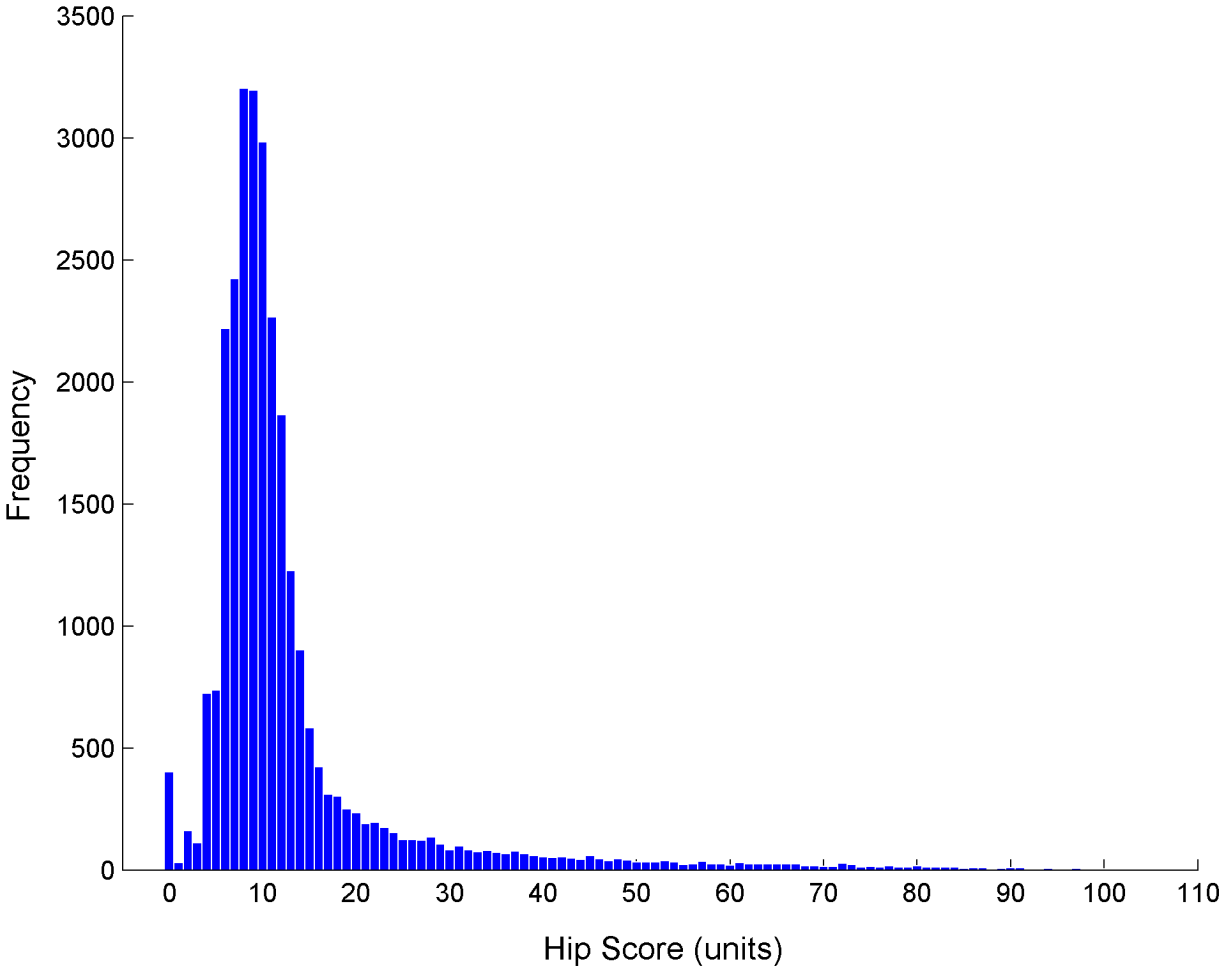
Distribution of hip score from all UK registered Labrador Retrievers. Dogs radiographed from 2000–2007 inclusive (where age at radiograph was above the stipulated 1 year old, recorded sex and coat colour were from the permitted classes male and female, and black, chocolate and yellow respectively, and scores within the defined boundaries of 0 to 106).

The distribution of hip scores (H) from radiographs taken and evaluated from 2000–2007 (Figure 1) had a mean of 13.22, (mode = 8, median = 10), a standard deviation of 12.29, CV = 0.93, and was highly skewed with coefficient of skewness of 3.39. Evaluation of radiographs taken of dogs aged 1, 2 and 3 years old comprised 90.6% of the scores. Given the progressive nature of the disease it would be expected that parameters would change over a lifetime and so the ‘full’ dataset used in the analyses described was limited to include only those dogs that were 1, 2 or 3 years (365–1459 days) old at the time of evaluation. This dataset (n = 25,243) had a mean of 13.00 (mode = 8, median = 10), standard deviation of 11.82, CV = 0.91, and coefficient of skewness of 3.44. There were 5,116 sires with a mean of 4.93 scored offspring per sire, and 12,719 dams with a mean of 1.98 scored offspring per dam, and 16,922 litters with a mean of 1.49 scored animals per litter. Generation interval (L) was calculated as 4.3 years. The mean coefficient of inbreeding (calculated over 5 generations of pedigree) was 0.041 (S.D. = 0.039), with 95% of the population <0.125 (the value for progeny of mating between two half-sibs that are otherwise unrelated).

### Lateral symmetry of gene expression

Under the BVA/KC scheme, left and right hips are scored separately and summed. The utility of analysis of the total score compared to a bivariate analysis of left and right scores (H_L_ and H_R_) depends upon the phenotypic and additive genetic variances, and the genetic correlation between left and right scores. The preliminary analysis of transformations as described in the Materials and Methods indicated that for individual left and right hip scores natural logarithm transformations of 1+H_L_ and 1+H_R_ were optimal. Very similar estimates of phenotypic variance (0.386±0.0074 and 0.366±0.0070) and heritability (0.30±0.036 and 0.27±0.035) were obtained for transformed left and right hip scores from two year old dogs. The genetic correlation between transformed left and right hip score was estimated with only small error but still could not be distinguished from 1, with a 95% confidence interval of [0.99,1.00]. In contrast, the environmental correlation between left and right hip score was 0.57 (±0.025). Given the similarity of estimates of heritability and phenotypic variances and the near perfect genetic correlation it was considered reasonable to simplify further analyses by using the transformed total score (log_e_(1+H), where H = H_L_+H_R_), since the benefit of bivariate analyses resulting in two EBVs for use in selection, or a differentially weighted sum would be negligible.

### Age related expression

To explore any need to model genetic parameters separately for H at different ages, a series of bivariate analyses were conducted for scores of 1, 2 and 3 year old dogs. Preliminary analysis of transformations of total score determined that within each year age group log_e_(1+H) was again the optimum. Models tested the hypothesis of genetic equivalence of transformed hip scores of 1, 2 and 3 year olds. Heritabilities (h^2^) and phenotypic variances (σ^2^_P_) were similar at all three ages (h^2^ = 0.33–0.34, σ^2^_P_ = 0.34–0.43) and genetic correlations between total scores at each age were not significantly different to 0.999 indicating the hypothesis of genetic equivalence of scores over this age range is reasonable. In contrast, a comparable bivariate analysis using an identical protocol suggested more dissimilar phenotypic variances and heritability estimates of dogs scored at one and five years old (1825–2189 days, h^2^ = 0.19, σ^2^_P_ = 0.50) although the genetic correlation of hip scores at the two ages remained high (0.95, and not significantly different from 1).

### Litter and dam effect on hip score

There are reports that hip score may be subject to litter and maternal effects [22] and so litter and dam random effects were first singly and then jointly added to a simple animal model to determine significance, and the results are shown in Table 1. Separate inclusion of either ‘litter’ or ‘dam’ as random effects in addition to ‘animal’ improves the fit compared to the ‘animal’ only model. However, inclusion of both ‘litter’ and ‘dam’ together in addition to ‘animal’ makes no significant improvement to fit compared to the addition of ‘litter’ alone. These results indicate that the data analysed contains insufficient records from repeated litters from dams to establish separate effects, or that the dam effects are small. An ‘animal’+‘litter’ model was used in subsequent analyses since it had a greater likelihood compared to ‘animal’+‘dam’.

**Table 1:** Model Log likelihoods.

Model	Effects	Log likelihood	Test statistic	P
1.	animal	898.77		
2.	animal+litter	928.00	58.46 c.f. Model 1	<0.001
3.	animal+dam	911.42	25.30 c.f. Model 1	<0.001
4.	animal+dam+litter	928.39	0.78 c.f. Model 2	>0.05
			33.94 c.f. Model 3	<0.001

Log likelihoods of a sequence of models fitting combinations of ‘animal’, ‘litter’ and ‘dam’ terms to log_e_(1+H), together with the test statistic for the likelihood ratio tests. The significance value, P, is obtained from comparison with χ^2^_1_.

### Genetic parameters and trends for total hip score (H)

Using the ‘full’ dataset described the heritability estimate of log_e_(1+H) was 0.35±0.016. The variation due to litter explained a fraction of 0.07±0.009 of the total variation, much smaller than the genetic. The total phenotypic variation was 0.375±0.0040. For comparison, when untransformed hip score was analysed using the same model the heritability and litter variance fraction were estimated at 0.50±0.018 and 0.06±0.009, and phenotypic variance was 153.1±1.8 (see later).

**Figure 2:**
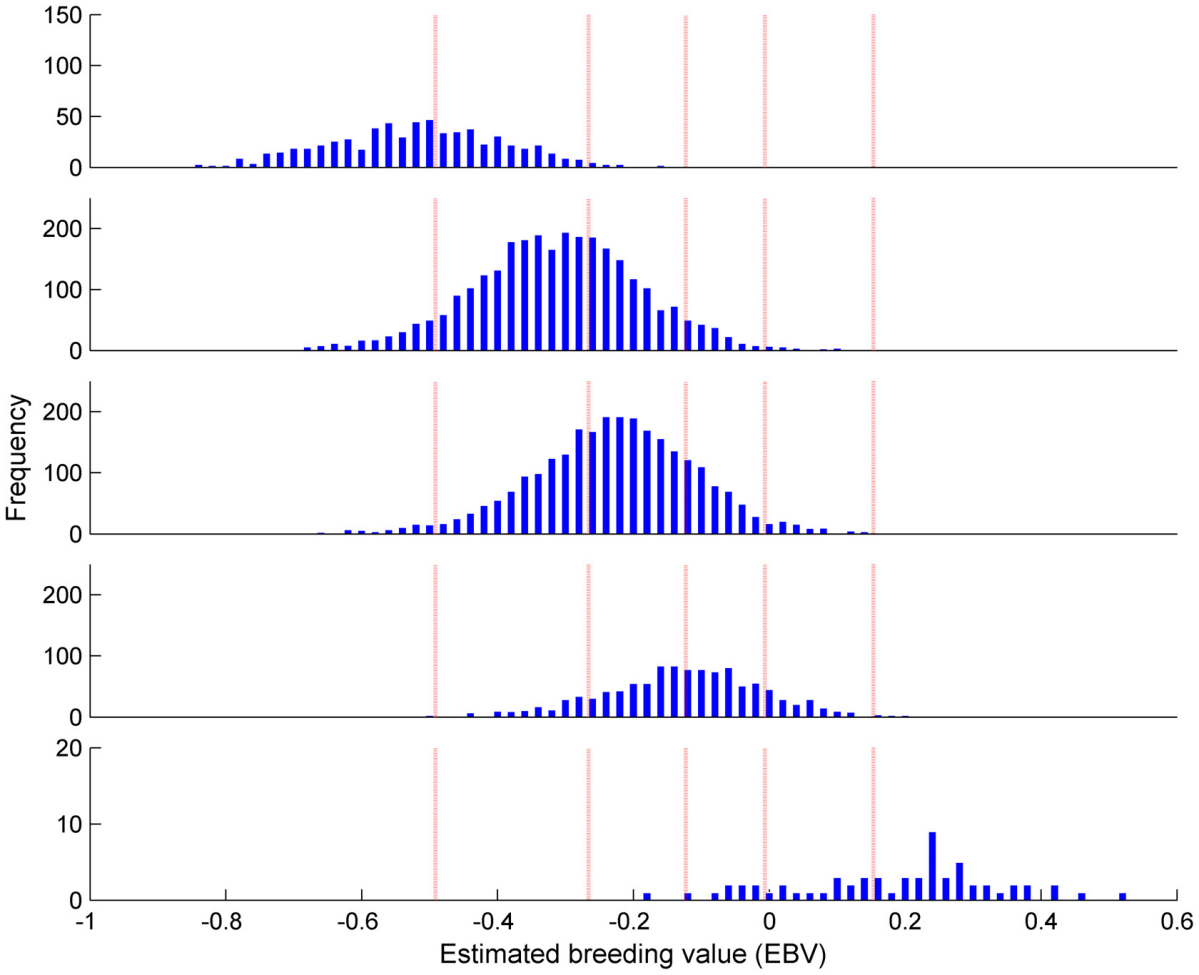
Distribution of EBV over phenotypic scores. Subsets defined by phenotypic scores H≤4, H = 8, H = 10, H = 13 and H≥37 respectively. These subsets represent the lowest (best) 5% (top), individuals with phenotypic values equal to the lower quartile, the median and the upper quartile, and the highest (worst) 5% (bottom). Vertical dashed lines indicate the 5^th^, 25^th^, 50^th^, 75^th^ and 95^th^ percentile for the EBV. EBV are obtained from analysis of log_e_(1+H).

Figure 2 shows the distribution of EBV, when evaluated on the log scale, of dogs with hip scores H≤4,  = 8,  = 10,  = 13, and ≥37 corresponding to the lower and upper 5% tails of the phenotypic values, the quartiles and the median. The overlapping of the distributions shows the large range of EBV that can occur within a single phenotypic hip score, for example a median score can be associated with EBV either in the best or worst 5%. This refinement of the genetic breeding value is one potential advantage of EBV over phenotype for providing predictions of risk in a breeding program to reduce hip dysplasia.

The mean and standard deviation for EBV of log_e_(1+H) and the observed hip score (H) according to year of birth are displayed in Table 2. Both mean EBV and mean H decrease with year of birth indicating that there is moderate progress against hip dysplasia, and that this is partially mediated by genetic improvement. The decline in H from 1996 to 2006 is 2.69 untransformed units, and regression of H on date of birth equivalent to a fall of 0.376 (±0.031) untransformed units per annum. For the same period EBV declined by a total of 0.155 transformed units, and regression on date of birth was equivalent to a decline of 1.36×10^−2^ (±0.0007) transformed units p.a. This trend in EBV between 1996 and 2006 is equivalent to a 1.4% decline in (1+H) year on year. Translating this estimated decline to observed H shows genetic progress has reduced hip scores by 13% from 1996 to 2006 birth years. This can be compared to the 20% decline observed phenotypically, which includes non genetic factors such as changes in nutrition and exercise regimes, and indicates that genetics has been the dominant factor in improvement. The estimate of total gain achieved in this period of 1996 to 2006 birth years of 0.155 transformed units is equivalent to 0.43 genetic standard deviations. The standard deviation of phenotypic hip score declines over progressive years of birth, consistent with a near constant CV of 0.9, and is indicative of a contraction of the ‘tail’.

**Table 2:** Hip score (H) statistics over year of birth.

Year of birth	n records	EBV		H	
		Mean	s.d.	mean	s.d.
1996	169	−0.160	0.232	14.04	12.40
1997	665	−0.167	0.249	14.59	13.78
1998	1576	−0.174	0.245	14.20	12.53
1999	2703	−0.195	0.239	13.40	11.55
2000	2967	−0.183	0.242	13.89	12.62
2001	3160	−0.201	0.247	13.70	12.76
2002	3465	−0.221	0.236	13.03	11.81
2003	3827	−0.240	0.241	12.88	11.91
2004	3443	−0.245	0.235	12.03	10.99
2005	2489	−0.267	0.234	11.41	9.92
2006	779	−0.315	0.246	11.35	9.87

Mean and standard deviation of EBV and phenotypic hip scores (H) according to year of birth for all records of dogs scored at ≥1 and <4 years old and between 2000 and 2007. EBV are obtained from analysis of log_e_(1+H).

### Accuracy of selection

The accuracy of an EBV in predicting the true breeding value, or genetic liability, at the time of selection is directly proportional to the rate of progress. However, the accuracy of an EBV will change over time as more phenotypic information becomes available on the individual or relatives, and so the lifetime profile of EBV accuracy against age will influence the overall utility of EBV and recommendations on how to use them. This profile was examined by comparing results obtained with the full data set to those obtained from a reduced dataset, where the data of the final cohort (779 dogs born in 2006) was set as missing. Estimates from analysis of the reduced data mimic the ‘nascent’ EBV for the 779 dogs born in 2006 which would have been predicted at birth as ½EBV_sire_+½EBV_dam_, i.e. prior to scoring and so without individual phenotypic records. The mean accuracy of nascent EBV for the 779 dogs born in 2006, equivalent to EBV for a ‘newborn’ dog, was 0.55 (S.D. = 0.061). The mean EBV accuracy of the 779 dogs born in 2006 calculated with their scores included in the data was 0.70 (S.D. = 0.023). The different distributions indicate a 1.27-fold improvement in EBV accuracy obtained from the presence of an individual phenotypic record. Mean accuracies of EBV for sires and dams of the 779 dogs born in 2006 using the reduced dataset were 0.78 (S.D. = 0.121) and 0.70 (S.D. = 0.095) respectively, approximating the accuracy of sire and dam EBV at the time of breeding within the current system. Therefore whilst a typical sire has a 1.1-fold higher accuracy than that expected after simply being scored itself, most likely due to information from previous offspring, the accuracy of a typical dam is not supplemented by additional information. Using the full dataset, the mean accuracy of sire and dam EBV was 0.80 (S.D. = 0.103) and 0.72 (S.D. = 0.077) revealing a relatively minor incremental improvement in accuracy of estimate from this additional litter, in the current system.

In the absence of EBV, any selection must be based upon the phenotypic score of the individual. In this case the accuracy of selection when selecting on the phenotypic scores of candidates is 0.59 (equal to h). This compares to the accuracy of 0.70 when using the EBV after scoring, indicating a potential 1.19-fold increase in progress − even if selection intensity were not to increase when the EBV was available (see Discussion). Choosing parents on the average of their phenotypic scores produces an accuracy for the new born of 0.42 (calculated as √½ h, which is optimistic since it *ignores all potential biases* from fixed effects in such a procedure!). Therefore the accuracy of the nascent EBV for a newborn of 0.55, calculated above, represents 1.31-fold increase in accuracy.

### Assessment of historical selection intensity applied

The results reported above may be used to calculate selection intensity (*i*) that has been applied against hip dysplasia since breeders will only have had access to a phenotype, and so rate of progress, *ΔG*, is given by *i h^2^ σ_P_ / L*. Substitution with the estimates for log_e_(1+H) of *σ_P_* = 0.61, *L* = 4.3, *h^2^* = 0.35, and *ΔG* = 1.36×10^−2^, gives *i* = 0.27; this is equivalent to only breeding from dogs and bitches in the top 85% of the distribution, assuming a Normal distribution of values.

**Figure 3:**
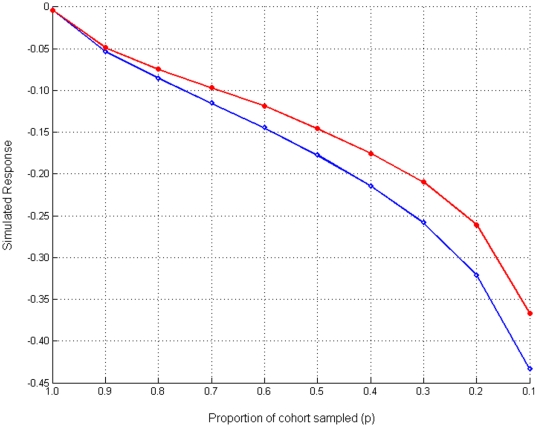
The simulated response to selection. Calculated as the difference in simulated generation mean EBV from parental generation mean EBV, due to selection from sampling within a cohort of dogs with the lowest *p* proportion phenotypic hip score (red line, closed circles) or EBV (blue line, open diamonds). Response is negative since lower EBVs indicate lower disease liability.

These calculations were tested using simulation of selection of dogs born in 2005 (n = 2489, 756 males, 1733 females). Figure 3 shows that whenever selection was practiced, i.e. *p*<1.0, the simulated response to selection indicated a greater response using EBV than phenotypic hip scores for every selection proportion, with the increment becoming larger as selection became more intense. The predicted responses in Figure 3 were used to provide a further estimate of the equivalent selection proportion being applied against hip dysplasia in the UK Labrador Retriever population. For the purpose of this comparison, the responses in Figure 3 need to be corrected for the 1.1-fold higher accuracy of a typical sire: since *ΔG* is directly related to accuracy but sires only contribute ½ the genes, the responses in Figure 3 need to be increased 1.05-fold. Furthermore responses in Figure 3 may be viewed as being equivalent to progress in a whole generation (with generation interval, L, of 4.3 years). The progress made in a single generation over the period 1996–2006 is 4.3×(1.36×10^−2^/1.05)∼0.056 transformed units. Having corrected for these two factors, the observed response can be seen to be equivalent to a selection proportion of ∼0.87 − in good agreement with the calculation above.

### Fixed effects on hip score

Analysis of the full data set indicated that males had a lower score compared to females of −0.042±0.0085 (P<0.05) on the transformed scale which is equivalent to approximately half a point when compared to females with a score of 10 (the median) and otherwise similar fixed and random factors. There was a small but detrimental trend (P<0.1) in hip score associated with the coefficient of inbreeding, equivalent to a quarter point increase for a median score of 10 (or 1.3 points for a score of 50) when comparing a coefficient of inbreeding of 0.125 to 0 (values obtained for offspring of half-sib and unrelated matings respectively). However, breeding from such close relatives is rare with less than 5% of animals resulting from a mating equivalent or closer than a half sib mating. Therefore avoidance of mating close relatives *per se* will have minimal impact upon the distribution of population scores.

**Figure 4:**
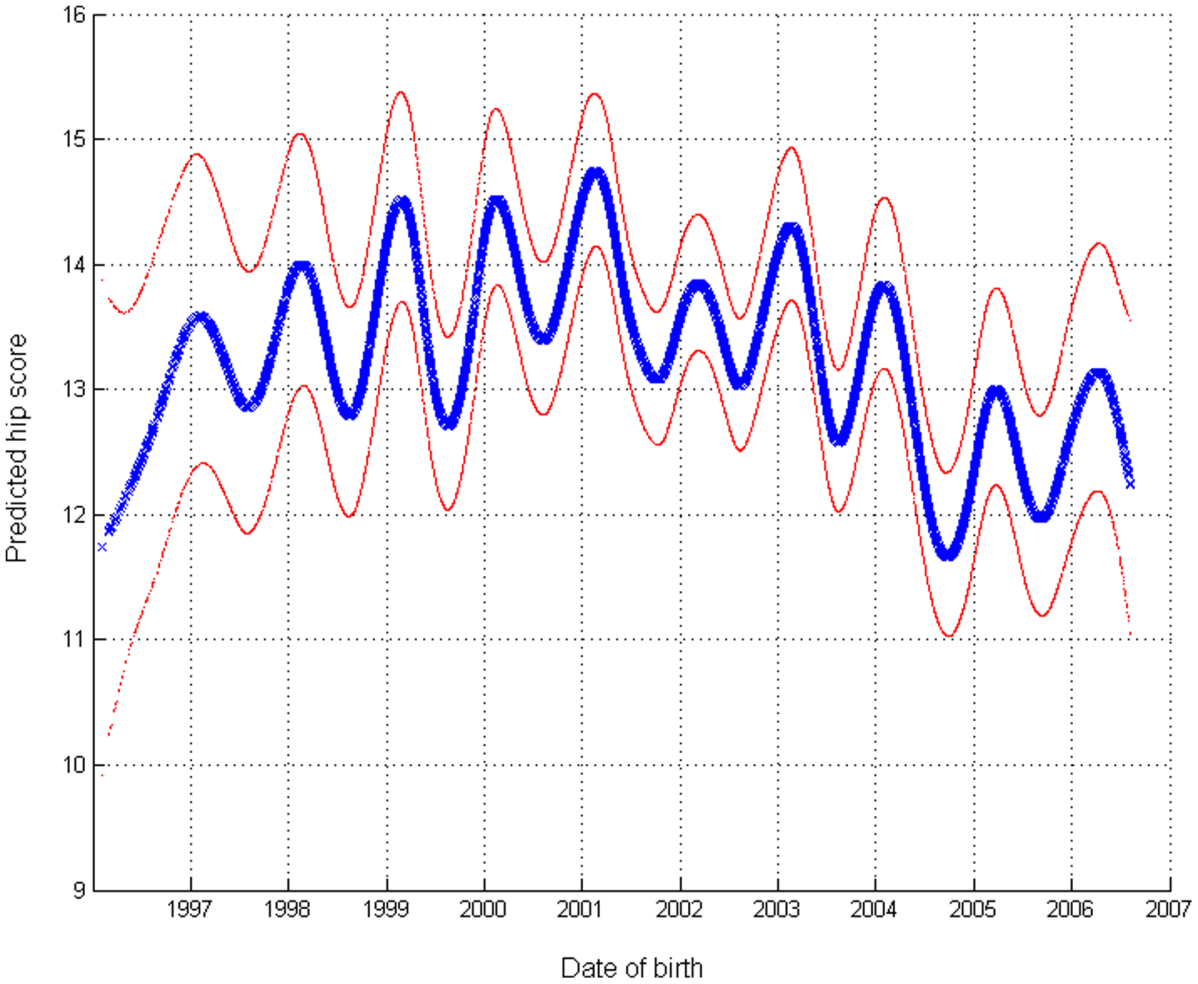
Predicted hip score over date of birth. Undulations within year indicate the seasonal effect on hip score. The blue line is the predicted value and the red lines indicate±1 standard error. Values shown were obtained from the fitted values for log_e_(1+H) by transforming back to the observed scale. The standard error curves were obtained by first adding ± s.e. to the fitted value on the transformed scale and then transforming back to the observed scale.

Whilst the pedigree accounted for genetic trend, the fitting of a smoothing spline for day of birth protected against any confounding that might have been present. Predicted values of hip score over smoothed splines of absolute day born show a definite cyclical pattern (figure 4) with scores elevated in winter and reduced in summer. This seasonal effect on hip score appears to have peaks and troughs varying between one and two points on the observed scale. The scale and timing of the seasonal effect on hip score is consistent with that reported previously in Labrador Retrievers [23].

The effect of age on hip score is detrimental with a predicted increase of just over a point from 12.8 at one year old to a plateau at 14.0 at about 2 ¼ years. However these differences are not large compared to the standard errors ranging from 0.5 to 0.8 on the untransformed grades. A trend in year of evaluation reflects either procedural change, including personnel, or changes in risk factors, since pedigree inclusion has accounted for any genetic trend in the data. There was no clear and consistent trend in the effect of year of evaluation over 2000–2007.

### Comparison of genetic evaluation of transformed and untransformed score

**Figure 5:**
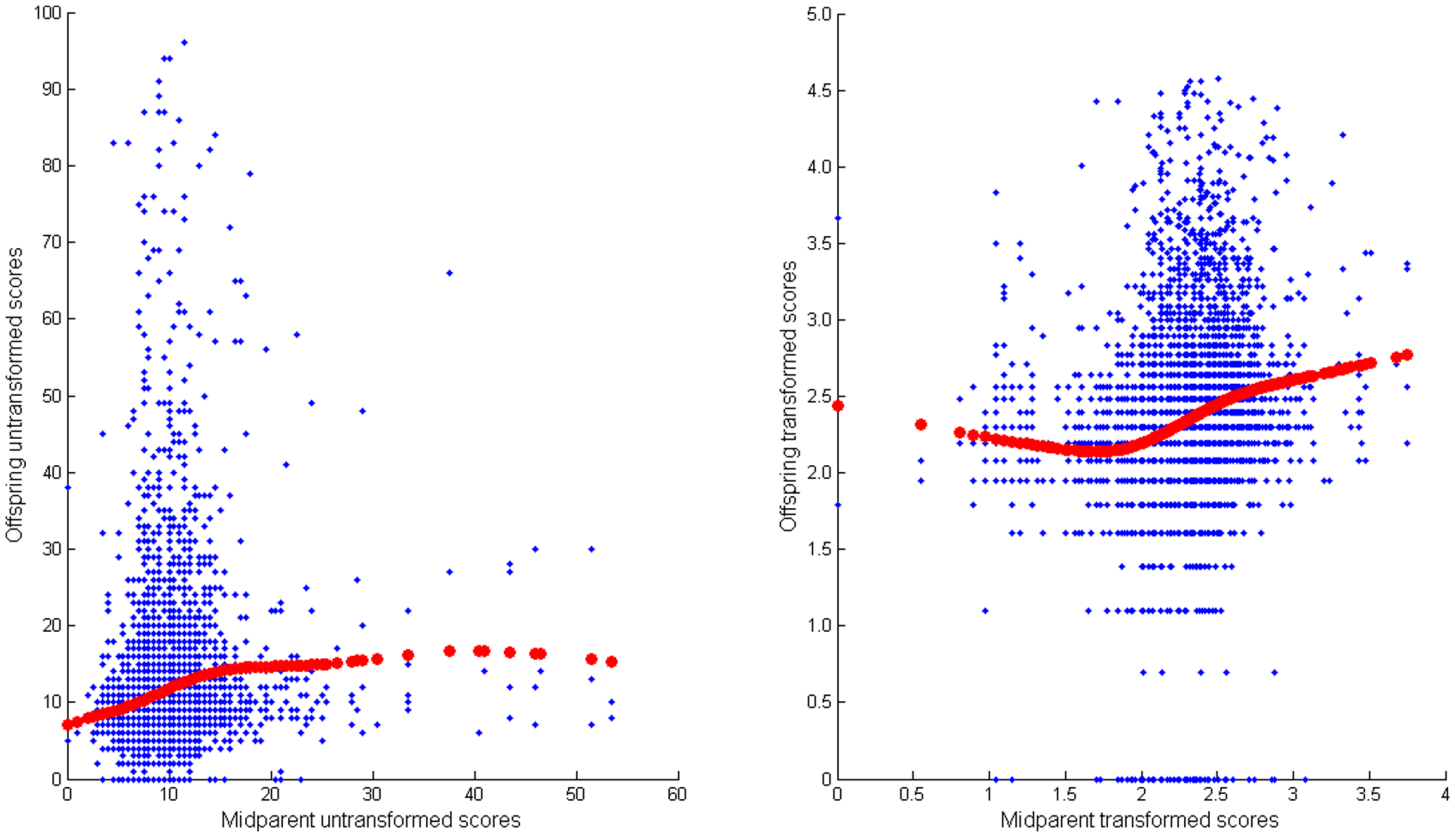
Relationship of offspring to midparent. Hip dysplasia scores (H) untransformed (left) and after transformation of individual scores to log_e_(1+H) (right). The points displayed are for the subset of 5115 records for which score data was available on the individual and both parents. The fitted values from regression on mid-parent are also shown using smoothing splines with 4 d.f. for smoothing the fitted curves (Genstat).

Given the relatively stable temporal trends in evaluation of hip dysplasia it was feasible to compare the relationship of scored offspring with the mean score of the parents, for both H and log_e_(1+H). For the transformed score the slope of the regression of offspring/mid-parent regression of transformed scores was consistent with the heritability estimate reported (b = 0.32±0.025) and yielded a reasonable fit over the distribution apart from the lower 5% (Figure 5). For the untransformed data the slope of the regression was 0.33, lower than the estimated heritability of 0.50 obtained from REML, with major deviations in the upper 5% of the tail. The relevance of this is discussed later.

### Genetic association of hip score with coat colour

To explore a possible genetic association of coat colour with hip score, coat colour was fitted as an additional fixed effect in the models described in the Materials and Methods. However there was no significant effect of colour on mean hip score (P>0.05) and the magnitude of the estimated genetic variance after adjustment for coat colour was only negligibly different from before adjustment. In conclusion there was no evidence of any genetic association between coat colour and hip dysplasia.

## Discussion

This study has shown that despite the large quantity of screening data provided by the BVA/KC hip scoring scheme, progress against hip dysplasia appears minimal; equivalent to that projected from avoiding only the worst 15% of animals for breeding. The introduction of EBV alone would be projected to increase the rate of progress by 19% through additional accuracy of selection even if selection intensity remained unchanged. Barring costly and painful surgery, hip dysplasia is incurable due to the development of osteoarthritis as a consequence of malformation, so genetic selection presents the only effective method of reducing the prevalence. Therefore, as hip dysplasia is one of the most serious diseases in larger breeds of dog, the need for the most efficient and effective genetic selection is clear.

The presented results have demonstrated that the availability of EBV through routine evaluations of the hip score data would hasten progress in alleviating the problem of hip dysplasia via increases in selective accuracy compared to selection based on phenotype alone. However the benefits of EBV extend beyond the simple comparisons of accuracy for a recently scored dog: (i) the EBV for an individual, unlike its phenotypic score, will further increase in accuracy over time by utilising all the available information and being updated as additional information becomes available e.g. from offspring or siblings; (ii) the EBV will provide predictors for those animals that do not have a phenotypic record hence increasing selection opportunities and intensity, which again enhances rate of improvement; (iii) the EBV will be available from the moment of birth for selection (although newborn littermates will have identical EBV) and, in this case, the accuracy (and hence rate of improvement) from using EBV increases by 31% compared to the parental average phenotype; (iv) the EBV will have been corrected for other fixed effects such as sex and age which bias phenotype as a predictor of genetic merit; and (v) it may be argued that taking account of a sustainable rate of inbreeding as well as disease prevalence would restrict the selection pressure that can be applied, however this only serves to place a greater emphasis on the accuracy of the selection that does take place. Finally with the availability of sequence [24] and dense canine SNP chips, the development of a genomic EBV (an EBV informed by additional information from dense SNP genotypes [25]) would help to distinguish littermates and further increase accuracy, increasing the potential rate of improvement, and might also lead subsequently to scientific benefits through identifying the major QTL. The intention is to make public the EBV for hip score for all KC registered Labrador Retrievers so that all these benefits may be realised.

The analyses of transformations for calculation indicated consistently that a logarithmic transformation was more suitable for the data, despite the observation of a higher estimate of heritability for H untransformed. Two important reasons argue for the use of the transformation. Firstly the Box-Cox analysis optimises transformation on the basis of implicit assumptions with the model; namely normality, lack of heterogeneity in subclass variance, and additivity of model terms, and whilst the procedure has been used here in the context of mixed models rather than a fixed model in which it was developed the underlying principles behind the optimisation may be assumed to hold. These implicit assumptions underpin substantial parts of quantitative genetic theory and therefore it seems a wise precaution to use the transformed scale. A second justification may be found in a more detailed examination of the additivity in a genetic context where similarity between offspring and parent is fundamental, and where in selection theory linearity in regression of offspring on mid-parent an important tenet. This examination was possible because of the relatively stable temporal trends for evaluation of hip score, which testifies to good quality control by the BVA. The implication is that focussing on the upper tail of the distribution alone is unlikely to have the benefit that may be anticipated in reducing the population mean, and that genetic progress needs to be generated by influencing selection within the wider population that is less extreme. Transformation alone does not alter this – the transformation is monotonic and so does not change ranking. However the more linear relationship between offspring and mid-parent will underpin a more predictable response to selection. The lack of linearity and monotonicity in the lower tail of the relationship for log_e_(1+H) is not influential as it affects only the lower 5% of the distribution and that part of the distribution which will always be selected. A possible explanation for the results concerning the lower tail may be lower precision of evaluation, for which there may be some support from the apparent excess of zero individual scores (see Figure 1).

A further caveat of the BVA/KC scheme arises from the possible under-reporting of extremely poor hip scores, since submission of the radiograph to the BVA is voluntary. In such cases, it would be hoped that a prospective hip score severe enough to warrant saving the cost of evaluation by the BVA panellists would dissuade the owner from using the dog in question for breeding. Directed removal of data from one end of the scale is expected to under-estimate heritability and consequently bias estimates of EBV; in particular sires with the poorer breeding values since they are expected to have more offspring with missing (and bad) records. However, selection progress from the existing data will still be expected, and potentially faster than predicted here as a result of the underestimation of sires with poorer breeding values. Therefore whilst submission of all radiographs would be better, the BVA/KC scheme remains of high quality, scoring a large number of dogs with the majority of breeding stock. Such biases are present in many recording schemes, for example in preferential treatment of cows in dairy breeding.

This study clarified that the total hip score (of both left and right hips) was the appropriate statistic for genetic evaluation since investigation showed that left and right hips had near identical genetic parameters. The optimum weighting for individual hips, in principle, favours the hip that is richer in genetic information and this is related to heritabilities and phenotypic variances for each hip. However the demonstration of near perfect genetic concordance across hips indicates that analysis of total hip score averages out the environmental influences that differentiate the individual left and right hip score (note the environmental correlation was only 0.57). Furthermore, given the extent to which genetic influences are shared by both hips (the genetic correlation was 0.999) recording the worst hip only will add bias by recording the hip that has suffered from the most extreme deleterious environmental impact. This was supported by additional analysis which indicated that EBV for a measure of mean hip score was a better predictor of both mean and worst hip score than EBV for worst hip score (see supplementary Material S1 and Table S1).

The rate of genetic improvement estimated in this study is modest, equivalent to 0.43 genetic standard deviations p.a.; lower than that reported in Swedish Rottweilers from 1992–2002 (0.67) [16], but higher than that in US Labradors from 1970–2005 (0.37) [17]. Comparison between the US and UK studies are more straightforward since they consider the same breed/gene pool and, given the low selection intensity observed in the UK, the disparity of progress may be due to the recording schemes since the more refined scale of the BVA/KC was estimated to be 1.6 fold more heritable than the 7 point OFA scheme. Comparison with Swedish Rottweilers however is across gene-pools, and the reported heritability for the 5 point FCI scheme was marginally greater though with greater standard errors. Thus, whether the superior rate of improvement reported by the Swedish study is due to breed, to recording scale or to greater selection pressure is unclear.

To date any selection against hip dysplasia in the UK will have been accomplished using phenotypic hip score, and the BVA endorses such practice by recommending breeding from dogs with scores clearly below the breed mean, which is currently 15 (http://www.bva.co.uk/public/documents/chs_hip_scheme_breed_mean_scores.pdf). However, it is clear that the BVA recommendations are only just short of being met, since a score of 15 corresponds to the 81^st^–82^nd^ percentile in our data, equivalent to avoiding only the worst 18–19% of animals for breeding – close to what is observed. At the current rate of progress (an improvement in mean EBV of 1.36×10^−2^ per annum), an ambitious but realistic target of a reduction in the median hip score from 10 to 5 would take over 44 years using phenotypic selection, or just over 37 years if selection was on EBV of dogs with a phenotypic hip score. Therefore, despite the improved accuracy of selection enabled by EBVs, there remains very slow selective progress against hip dysplasia. Even breeding below the median phenotype, i.e. best 50% of animals, would have resulted in progress over 2.5 fold greater than has been observed over the period 1996–2006. The selection intensity from breeding from the best 50% of scored animals using EBVs would be over 3 times higher than that achieved over 1996–2006 where breeding from dogs with scores less than the current breed mean was the guideline. These figures indicate that, even with the improved efficiencies afforded by EBV, adequate selection pressure is also vital in improving progress against hip dysplasia and that this could be achieved with more challenging guidelines.

## Materials and Methods

### BVA/KC hip score scheme

The BVA/KC hip-scoring scheme is voluntary and, to ensure skeletal maturity, restricted to dogs over a year old [23]. There is no upper age limit restricting participation, but dogs may only be scored once. Eight of the nine features considered in the scheme are scored 0–6 for each hip, and the ninth is scored 0–5 for each hip (a zero score indicating no signs of dysplasia in both cases) resulting in a score out of 53 for each hip, or 106 in total, which is considered to describe the general condition of the dog's hip joints. The nine features are scored on the detectable laxity of the joint, bone formation and the degree of any exostosis (abnormal bone growth) and wearing. A more detailed description of the scoring criteria is given by Gibbs [12].

### Dog and pedigree data

Data on left, right and total hip score were obtained from the Kennel Club and restricted to Labrador Retrievers scored between 2000 and 2007 inclusive, since this restriction helped to minimise any diagnostic drift. Other data obtained for the same dogs were their sex, date of birth and date of radiograph. Data were also restricted to those records where sex was explicitly stated as male or female; coat colour was stated to be one of the ‘permitted’ colours (i.e. black, chocolate and yellow only); and the hip score was within the defined boundaries of 0 to 106. In the ‘full’ dataset the age at scoring was limited to within the boundaries of ≥1 and <4 years old (365 to 1459 days old inclusive) since over 90% of information available came from this relatively narrow age range and there would be an expectation of changing parameters over a wider period given the progressive nature of hip dysplasia. The resultant data set contained 25,243 single records of hip score from radiographs taken between 2000 and 2007 of dogs born between 1996 and 2006. The data were unevenly divided between the sexes with many more females than males scored, 75% to 25%. The fractions of each colour were close to those in the KC pedigree (black, 55 in study v 52% in KC; chocolate, 20 v 13%; yellow, 26 v 29%) indicating no obvious selective bias or stratification on the basis of coat colour. Records were distributed roughly equally over year of evaluation (max 2006 = 14.8%, min 2007 = 9.8%). The data records were linked to the KC Labrador pedigree database using their unique registration number. All ancestors of the dogs with hip score data were traced back to the founding generation, i.e. where the sire and dam are unrecorded, or to a maximum of four generations (great, great-grandsires/dams). As a result, the pedigree used in the initial analysis comprised 62,683 animals in total with 99.9% of grandparent identities of dogs with scores known. The coefficients of inbreeding were calculated using this restricted pedigree. Mean and standard deviation of inbreeding coefficients for dogs with data were 0.041 and 0.039 respectively.

### Statistical Analysis

Mixed linear models were fitted to the data using ASREML [26] to estimate variance components for the hip score (H). Examination of the appropriate scale for analysis was undertaken given the skewed distribution of H (Figure 1). Preliminary analyses considered the family of power transformations [27] on 1+H, where the 1 was added to avoid the logarithm of zero − here without loss of generality, natural logarithms were used at all times. Transformations were assessed using the family of power transformations [28] (results not shown) with preference given to more readily interpretable scales of untransformed, square root, logarithmic, 1/square root and reciprocal. The optimum transformation was found to be log_e_(1+H) and this form was used as the basis of all further analyses, however the final model was also fitted on the untransformed scale for purposes of comparison.

After resolving the scale for analysis, further preliminary models tested the hypothesis of genetic equivalence of left and right hip scores and the total hip scores of 1, 2 and 3 year olds. Subsequently, the significance of ‘litter’ and ‘dam’ random effects were determined using likelihood ratio tests. The full form of the linear model was as follows:
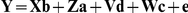
where **Y** is the vector of observations, **V, W, X** and **Z** are known incidence matrices, **b** is the vector of fixed effects, **a** is the vector of random additive genetic effects with the distribution assumed to be multivariate normal (MVN), with parameters (0, σ^2^_a_ **A**), **d** is the vector of random dam effects distributed MVN with parameters (0, σ^2^_d_ **I_dam_**), **c** is the vector of random litter effects with the distribution assumed to be MVN, with parameters (0, σ^2^_c_ **I_litter_**), **e** is the vector of residuals distributed MVN with parameters (0, σ^2^_e_ **I**). **I** represents an identity matrix of an appropriate size, **A** is the additive genetic relationship matrix, and σ^2^ denotes the variance of each of the respective random effects. The fixed effects included in the model were: sex, inbreeding coefficient, age in days at evaluation, birth date measured as days since 1^st^ Jan 1990, and year of evaluation. Age in days and absolute day of birth were fitted with random smoothing splines to model the temporal trends. Note that date of evaluation in days is the sum of birth date and age and cannot be fitted as an independent term.

### Accuracies of EBV of parents and of individuals at birth and after scoring

Accuracies (*r*) of EBV were calculated as:
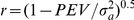
where PEV is the prediction error variance of each breeding value and σ^2^_a_ is the estimated additive genetic variance obtained from the mixed model analysis. ASReml provides both the estimates of EBV and their associated PEV. Repeat analysis of the ‘reduced’ dataset using the final model described provided ‘nascent’ EBV with associated PEV for the 779 dogs born in 2006 for analysis of accuracies.

### Assessment of potential progress using simulation of selection on EBVs and phenotypes

Simulation of selection for a single generation using both EBV and phenotypic hip score (H) was undertaken and the responses compared. The simulation produced 1000 matings between sires and dams sampled from the cohort born in 2005. Sires and dams were randomly selected with replacement from the lower x% of the distribution of either 1) phenotypic total hip score (H), or 2) EBV produced from the reduced dataset. The value of x varied from 90 to 10 in steps of 10. Each of these 18 combinations was replicated 1000 times. For each combination the difference in the overall mean of simulated nascent EBV of the offspring (½ EBV_sire_+½ EBV_dam_) and the mean EBV of all animals born in 2005 provided an estimate of response to selection after a single generation of selection.

## Supporting Information

Material S1 Additional analysis indicating superiority of mean over worst hip score.
(0.03 MB DOC)

Table S1 The correlation of nascent EBVs with observed phenotypes for log-transformed AVRG and WORST, calculated for the 779 dogs born in 2006.
(0.03 MB DOC)
