# Supplementary material for: Genetic Evaluation of Hip Score in UK Labrador Retrievers
Source: PLoS One. 2010 Oct 22;5(10):e12797. doi: 10.1371/journal.pone.0012797 (PMC2962628; doi:10.1371/journal.pone.0012797)
Supplement: Material S1 — Additional analysis indicating superiority of mean over worst hip score. (0.03 MB DOC) [file pone.0012797.s001.doc]

*Predictive ability of total and worst hip scores*

As noted in the introduction, recording schemes have different approaches in either recording the condition of both hips or the worst hip only. Our analysis of left and right hip score demonstrated near perfect genetic correlation across both hips, implying that recording the worst hip adds bias by recording the hip that has suffered from the most extreme deleterious *environmental* impact. Therefore the relative predictive value of EBVs for worst hip and average hip score was tested for the 779 dogs born in 2006, which were the final cohort in the full dataset. In this analysis AVRG = 1+½H_L_+½H_R_ was used rather than 1+H since the scale was then more comparable to WORST = 1+max(H_L_,H_R_). Nascent EBV (½ EBV_sire_ + ½ EBV_dam_) for both log-transformed AVRG and log-transformed WORST were obtained for the 2006 cohort by analyses of the reduced dataset, i.e. excluding all phenotypes for the 2006 cohort, using the full model as described in Materials and Methods. The predictive ability of the nascent EBV for the cohort was then compared with both the log-transformed AVRG and log-transformed WORST phenotypes.

Simple correlations between nascent EBV and the transformed AVRG and WORST phenotypes are shown in Table S1. Results from Table S1 are indicative of superiority of the EBV for mean hip score over EBV for worst hip score at predicting *both* AVRG and WORST phenotypes. However these correlations are unadjusted for the fixed effects.

To investigate the predictive power of the EBVs more thoroughly, a further regression analysis was conducted on the phenotypes. A regression model was fitted in turn to log_e_(AVRG) and log_e_(WORST) with covariates accounting for sex and age in days at screening, together with either (i) EBV_AVRG_, or (ii) EBV_WORST_, or (iii) both EBV_AVRG_ and EBV_WORST_. Comparing (iii) with (ii), the addition of EBV_AVRG_ to the model significantly improved the fit for both log_e_(AVRG)and log_e_(WORST) (P<0.01 and P<0.05 respectively). However there was no improvement of fit (P>0.05) when comparing (iii) with (i). This provides evidence for the sub-optimality of calculating EBV from the worst hip score compared to the mean or total hip score.
