# Supplementary material for: Genetic Evaluation of Hip Score in UK Labrador Retrievers
Source: PLoS One. 2010 Oct 22;5(10):e12797. doi: 10.1371/journal.pone.0012797 (PMC2962628; doi:10.1371/journal.pone.0012797)
Supplement: Table S1 — The correlation of nascent EBVs with observed phenotypes for log-transformed AVRG and WORST, calculated for the 779 dogs born in 2006. (0.03 MB DOC) [file pone.0012797.s002.doc]

Table S1. The correlation of nascent EBVs with observed phenotypes for log-transformed AVRG and WORST, calculated for the 779 dogs born in 2006.

|  | AVRG | WORST |
| --- | --- | --- |
| EBV_AVRG_ | 0.231 | 0.223 |
| EBV_WORST_ | 0.221 | 0.213 |
